# Correlations and comparisons of teacher expectations achievement motivation academic achievement and creativity

**DOI:** 10.3389/fpsyg.2025.1516405

**Published:** 2025-08-21

**Authors:** Jun Hu, Shan Qian

**Affiliations:** ^1^School of Marxism, Hangzhou Normal University, Hangzhou, Zhejiang, China; ^2^School of Public Administration, Hangzhou Normal University, Hangzhou, Zhejiang, China

**Keywords:** teacher expectations, academic achievement, psychological effects, controlled experiment, achievement motivation

## Abstract

**Introduction:**

Teacher expectations are believed to play a critical role in shaping students’ academic achievement and creativity, yet the underlying psychological mechanisms remain insufficiently understood. This study investigates the mediating role of achievement motivation in the relationships between teacher expectations, academic achievement, and creativity among junior high school students, aiming to provide empirical insights for effective educational practices.

**Methods:**

A controlled experimental design was employed to examine the psychological effects of teacher expectations. The sample consisted of 583 students from urban and township junior high schools. Teacher expectations were measured using the Teacher Expectation Scale. Academic achievement was assessed via final examination scores in Chinese, mathematics, and English. Achievement motivation was evaluated with the Achievement Motivation Scale, and creativity was measured using a standardized creativity assessment. Data were analyzed using analysis of variance (ANOVA), correlation analysis, and structural equation modeling (SEM).

**Results:**

Teacher expectations had a significant positive effect on academic achievement. Achievement motivation partially mediated the relationship between teacher expectations and academic achievement, and fully mediated the relationship between teacher expectations and creativity. The mediation models demonstrated strong fit, confirming the central role of motivation in translating teacher expectations into student outcomes.

**Discussion:**

The findings confirm that teacher expectations influence both academic performance and creative development through motivational pathways. Achievement motivation serves as a key psychological mechanism that links external educational beliefs to internal student drives. These results support the implementation of teacher training and classroom strategies that foster positive expectations, thereby enhancing students’ motivation, academic success, and creative potential. This study contributes to the growing body of evidence advocating for supportive and expectancy-rich learning environments in secondary education.

## 1 Introduction

Teacher expectations, as a key psychological variable in the educational process, have long been regarded as an important factor affecting students’ academic performance. There are two important limitations in the exploration of the mechanism of action in existing research: firstly, most studies adopt relevant designs, which make it difficult to establish a causal relationship between teacher expectations and student academic achievement; Secondly, empirical testing of intermediary mechanisms, such as the role of achievement motivation, is still insufficient. This study adopts a controlled experimental design to systematically examine the psychological impact and mechanism of teacher expectations on academic achievement of junior high school students (Tao et al., 2022). The experiment directly tests the causal effect of teacher expectations by manipulating the level of teacher expectations (high/low) and controlling for other teaching variables. At the same time, introducing achievement motivation as a mediating variable to reveal the pathway of “teacher expectations → achievement motivation → academic achievement.” This design not only compensates for the shortcomings in causal inference in related research, but also provides a deeper analysis of the psychological mechanisms underlying the impact of teacher expectations. The study selected middle school students as the subjects, mainly based on two considerations: firstly, middle school students are in a critical period of self-concept development and are particularly sensitive to teacher evaluation; Secondly, the academic performance during this stage plays an important predictive role in the subsequent educational trajectory of students. By using standardized final exam scores in Chinese, mathematics, and English as the outcome variable and combining them with the mediation measurement of achievement motivation scales, this study will provide more rigorous empirical evidence for the theoretical model of teacher expectations (Prasetyo et al., 2021). This study aims to address the above issues through a rigorous experimental design, and its scientific and practical significance is as follows: using randomized controlled experiments, directly manipulating teacher expectations (high/low), controlling for other teaching variables, and for the first time testing the role of teacher expectations at the causal level. Introducing achievement motivation as a mediating variable, revealing the pathway of “teacher expectations → achievement motivation → academic performance,” and filling the gap in mechanism research. The middle school student group is in a sensitive period of self-concept development, and teacher evaluation has a profound impact on their learning confidence and long-term educational trajectory (such as their choice of further education). The research results can provide a basis for educational practice, help teachers reduce unconscious bias, optimize teaching interaction, and promote educational equity.

## 2 Literature review

Teacher expectations play a crucial role in students’ academic development. Existing research indicates that they not only directly influence academic achievement but may also indirectly affect creativity and motivation through psychological mediators (Halimi et al., 2021). In particular, during junior high school, achievement motivation may serve as a key mediating factor between teacher expectations and student outcomes. Studies have also shown that improvements in self-regulation and motivational levels significantly enhance academic performance (Yan et al., 2020), further highlighting the importance of psychological factors in educational contexts. However, the mechanisms through which teacher expectations influence academic achievement and creativity via achievement motivation require further investigation. This study aims to examine the mediating role of achievement motivation in the relationships among teacher expectations, academic achievement, and creativity, providing empirical support for effective educational practices. In recent years, researchers have begun to focus on the boundary conditions of expected effects. Some scholars suggest that teacher expectations, as well as how teachers perceive parental expectations, act as intermediate factors between past academic performance and key aspects influencing learning in students with specific learning disabilities (SLD) (Núez et al., 2022). Notably, the impact of time stress differed significantly based on students’ levels of busyness: for busy students, it decreased grades in course exams (excluding experiential learning), whereas for less busy students, it actually increased their grades. These findings highlight the importance of educators recognizing that not all course activities contribute equally to student learning. When easier activities unrelated to learning are rewarded with more credits, overall learning may be compromised. The impact of academic satisfaction on diminishing student knowledge acquisition warrants further exploration, given its considerable influence on learning outcomes (Petrow, 2022). Teuber et al. (2023) investigated how parental involvement in children’s academic difficulties, either through supportive guidance or controlling behaviors, influenced their academic performance over time. Furthermore, they addressed methodological concerns by comparing the Random Intercept Cross-Lagged Panel Model (RI-CLPM) with the conventional Cross-Lagged Panel Model (CLPM) (Teuber et al., 2023). Cheng et al. (2023) investigated how academic delay, motivational beliefs, and academic performance interrelate in online science courses. Through learning analysis and multi-level modeling, they identified two components of academic delay: habitual and instantaneous factors. Their research revealed that, when motivational beliefs are taken into account, habitual delay has a more pronounced effect on academic performance compared to instantaneous delay (Cheng et al., 2023).

This study aimed to examine the correlations and comparisons among teacher expectations, achievement motivation, academic achievement, and creativity in junior high school students. The research addressed the following questions: how do teacher expectations influence students’ academic achievement and creativity? What role does achievement motivation play in mediating these relationships? It was hypothesized that teacher expectations positively affected academic achievement directly and indirectly through achievement motivation, while their impact on creativity was mediated entirely by achievement motivation. The variables under investigation—teacher expectations, measured via perceived behaviors and attitudes; achievement motivation, assessed through pursuit of success and avoidance of failure; academic achievement, indicated by standardized exam scores; and creativity, evaluated through vocabulary test performance—formed the theoretical backbone of this analysis, building on prior work to clarify their interrelations.

## 3 Research methods

This study adopted a controlled experimental design to directly test the causal effect of teacher expectations on student academic achievement. The controlled experimental design accurately evaluates the independent impact of teacher expectations by manipulating the level of teacher expectations and controlling other teaching variables, making up for the shortcomings of related studies in causal inference. The intervention subjects were teachers, who were induced to have high or low expectations by providing them with false information about their students’ abilities to observe their effects on students’ academic achievement. The study randomly assigned teachers to a high expectation group or a low expectation group. Teachers in the high expectation group were told that their students had high learning potential, while teachers in the low expectation group were told that their students had low potential. The intervention lasted for one semester, during which the interactions between teachers and students were recorded and analyzed to evaluate the impact of teacher expectations on students’ academic achievement.

### 3.1 Data collection

This study adopts a controlled experimental design to directly test the causal effect of teacher expectation level (high/low) on students’ academic performance by manipulating it, and analyzes the action path of achievement motivation based on the mediation model. The data collection process is divided into a pre-test stage and a formal test stage. The data sources include the Teacher Expectation Scale, Achievement Motivation Scale, Creativity Test and Academic Performance. In the pre-test phase, 356 questionnaires were randomly distributed to test the appropriateness of the scale and the effective response ratio, providing a basis for the formal test. In the formal phase, with the assistance of the uniformly trained examiner, the test was organized and administered simultaneously in sample schools in cities and towns. The Teacher Expectation and Achievement Motivation Scales were filled in paper form, and students answered anonymously and the examiner collected them uniformly. The creativity assessment was conducted on-site by a master’s student in psychology using the Torrance Creativity Vocabulary Test. The duration of a single test was controlled to 45 min to ensure operational consistency. The academic performance data was provided by the school’s Academic Affairs Office with the original scores of the final exams of three main courses. The research team standardized the *Z* scores according to the class dimension to eliminate the inter-class effect. During the data collection period, research ethics were strictly observed. All participants signed informed consent in advance, and the questionnaire data were stored anonymously with numbers to ensure student privacy and data confidentiality. The participants were divided into two parts: preparatory study participants and formal study participants, both of which were obtained using cluster random sampling method. Participants: Emphasize the active participation of the research subjects and demonstrate respect for them; Subjects: Emphasize the research subject as the object of study, implying that the research subject is in a relatively passive position. The total population of this study is junior high school students in cities and towns. The sampling process adopts a stratified random sampling method, first stratified by city and town, and then randomly selecting schools and classes within each stratum. This method ensures the representativeness of the sample in terms of city and town distribution and different grade distribution, and avoids sampling bias. The sample size was determined through statistical power analysis to ensure sufficient statistical power to detect the impact of teacher expectations on academic achievement.

#### 3.1.1 Preliminary study subjects

The breakdown of participants can be found in Table 1. The preliminary study participant data presented in Table 1 is mainly used for the following purposes:

**Table 1:** Sample structure of preliminary study.

Grade	Urban and rural		Total number of people
	City	Township	
First grade of junior high school	58	58	116
Second grade of junior high school	69	60	129
Third grade of junior high school	65	46	111
Total number of people	192	164	356

Sample representativeness test: Verify the effectiveness of the sampling plan (urban–rural ratio, grade distribution, etc.) to ensure that the sample structure matches the characteristics of the research population.

Methodology preparation: Test the feasibility of the data collection process, evaluate the quality of questionnaire completion and the proportion of invalid questionnaires (98.6% effective rate).

The sampling design in Table 1 adopts different grades (Grade 1 to Grade 3) and different regions (cities/towns), mainly based on the following research considerations:

##### 3.1.1.1 Consideration of grade differences

Developmental differences: the third year of junior high school is a critical stage for the cognitive and psychological development of adolescents, and the teacher expectation effect may change with the age of students (such as a decrease in sensitivity of junior high school students to teacher expectations).

Differences in academic stages: the difficulty of courses and the pressure of further education vary among different grades (such as facing the middle school entrance examination in the third year of junior high school), which may moderate the strength of teachers’ expectations.

Control grade variables: avoid research results being dominated by specific grade characteristics and improve the generalizability of conclusions.

##### 3.1.1.2 Consideration of urban–rural differences

Differences in educational resources: there are systematic differences in teachers, facilities, and other aspects between urban and rural schools, which may affect the formation and transmission mechanism of teacher expectations.

Cultural environment differences: there may be differences in the level of emphasis on education and students’ self-awareness between urban and rural families, which may interact with teacher expectations.

The 356 junior high school students presented in Table 1 are a pilot study, and these participants are pre study prediction samples selected through cluster random sampling from urban/township junior high schools.

#### 3.1.2 Formal study subjects

Select one junior high school from each city and township for a questionnaire survey. Five hundred ninety-five questionnaires were distributed, and after excluding invalid questionnaires, we received 583 valid questionnaires, indicating a solid response rate of 98%. The distribution of participants can be viewed in Table 2. In the study of the impact of teacher expectations on students’ academic achievement, the selection of urban and rural schools as research environments is based on rigorous theoretical and methodological considerations, especially when experimental methods are used. This design has important scientific basis: (1) to test the robustness of teacher expectation effects in different educational resource environments; (2) identify situational factors that may regulate the expected transmission process (such as family support levels); (3) improve ecological validity through natural field experiments and avoid the artificial nature of laboratory research.

**Table 2:** Sample structure of formal studies.

Grade	Area		Total number of people
	City	Township	
First grade of junior high school	57	114	171
Second grade of junior high school	135	61	196
Third grade of junior high school	126	90	216
Total number of people	318	265	583

The basis for predicting the sample size of research (*N* = 359): typically, at least 10–20 participants are required for each scale item. If teachers expect the scale to contain 30 items, at least 300 participants (30 × 10) are needed; the confirmatory factor analysis (CFA) for reliability and validity testing suggests a sample size of ≥200, with half urban and half rural to ensure the effectiveness of group comparisons; the invalid questionnaire buffer has a preset rejection rate of 10% (expected retention of 359 responses ≈ 320 responses), and the actual retention of 356 responses meets expectations.

### 3.2 Academic achievement measurement indicators

This study uses the objective scores of the subjects’ final exams in Chinese, mathematics, and English for the semester to represent their academic achievements. Due to the participants coming from different schools and classes, the final exam scores of Chinese, Mathematics, and English were standardized by class in the study, and the total score of the standardized scores in the three subjects was used as the academic achievement indicator for the participants (Gopal et al., 2021; Francisco and Celon, 2020; Papageorge et al., 2020). In this study, the classification of the two types of students is not based on objective abilities, but on the subjective expectations of teachers (measured through the Teacher Expectation Scale). Advantageous students refer to a group of students whose academic performance is positively expected by teachers; disadvantaged students refer to a group of students whose academic performance is negatively or neutrally expected by teachers.

### 3.3 Teacher expectancy perception assessment scale

The Teacher Expectancy Perceived Scale (TECS) consists of 22 items, divided into two dimensions: expected behavior and expected attitude. Among them, expected behavior refers to the teacher’s perceived way of doing things, which includes 14 items, such as “the teacher always encourages me to ask him/her at any time if I do not understand”; The scale employs a 5-point rating system, where scores range from 1 to 5, indicating “completely disagree” to “completely agree,” respectively. Within the scale, 10 items necessitate reverse scoring (Van der Spoel et al., 2020). Among them, the chi square degree of freedom ratio is calculated according to the Equation 1:


(1) $$\mathrm{Chi}\;\text{square degree of freedom ratio}=\chi^{2}/\mathrm{df}$$

Within the statistical analysis, *x*^2^ represents the chi square value, and df represents the degree of freedom.

### 3.4 Torrance Creative Thinking Vocabulary Test

The Torrance Creative Thinking Vocabulary Test consists of seven items that assess various aspects of creative thinking. These include questions about predicting reasons and outcomes, enhancing products, unconventional uses, posing unusual questions, and demonstrating logical imagination (Bardach et al., 2022). The analysis of the results obtained from untrained raters indicates that after carefully studying the scoring guidance, they can reach an acceptable level of reliability, with an average correlation between the two being higher than 0.91. The actual testing time for the Torrance Creative Thinking Vocabulary Test is 45 min.

### 3.5 Academic achievements

Regarding the collection of student academic achievement data, considering the delayed effect of teacher expectations and the different emphasis on distinguishing between main and sub subjects in actual teaching, the final exam scores (Chinese, mathematics, English) of the subjects were selected as the original academic scores (Orland-Barak and Wang, 2021).

### 3.6 Data analysis procedures

The data analysis of this study was carried out strictly according to the research hypothesis structure. First, SPSS 26.0 was used to perform normality tests and descriptive statistics on all variables, to exclude extreme values and missing data, and to perform *Z*-standardization on academic performance by class. Second, Pearson correlation analysis was used to explore the preliminary correlation between teacher expectations, achievement motivation, academic performance and creativity. To further test the hypothesized relationship and mediation path, AMOS 24.0 was used to construct a structural equation model, setting teacher expectations as the independent variable, achievement motivation as the mediating variable, academic performance and creativity as the dependent variables, and the maximum likelihood estimation method was used to estimate the path coefficient. The significance of the mediation effect was calculated by the Bootstrap method (5,000 resampling) with a 95% confidence interval. Model fit indicators included *χ*^2^/df, RMSEA, CFI, TLI and IFI to evaluate the degree of fit between the model and the data. All tests were two-sided, with *p* < 0.05 as the significant standard. To verify the effectiveness of the intervention effect, a baseline balance test was also conducted on the high and low expectation groups (all variables *p* > 0.20) to ensure that the experimental operation would not be interfered by the initial differences. The control experiment design generates grouped data by manipulating the teacher’s expected level (high/low), and subsequent analysis will combine correlation and causal inference methods: ANOVA and structural equation modeling to verify variable relationships and mediation pathways, and Bootstrap method to test the significance of mediation effects.

## 4 Result analysis

This section conducts analysis based on research hypotheses (H1–H3): H1 tests the direct relationship between teacher expectations and academic performance; H2 verifies the mediating role of achievement motivation between teacher expectations and academic performance; H3 explores the mediating pathway of achievement motivation on teachers’ expectations and creativity. The analysis covers variable characteristic description (4.1–4.2), variable correlation (4.3), and structural equation modeling test (4.4) in sequence.

### 4.1 Characteristics of teacher expectations

#### 4.1.1 Overall characteristics of teacher expectations

The scores for each dimension of teacher expectations are shown in Table 3. On the 5-level scale, the overall expected level of teachers is above average (*M* = 3.34, SD = 0.63). From the mean and standard deviation of the three teacher expectations, it can be seen that the scores of the three teacher expectations, from low to high, are: ability support, emotional preference, and learning requirements. The analysis of variance for repeated measurements of the three dimensions of teacher expectations revealed significant overall differences among the three types of teacher expectations (Wilks’ *Λ* = 0.572, *p* < 0.001). The results of further *post hoc* multiple comparisons indicate that teachers’ ability support for junior high school students is significantly lower than emotional preference and learning requirements (*p* < 0.001), and emotional preference is also significantly lower than learning requirements (*p* < 0.01).

**Table 3:** Description of teacher expectations and *post hoc* testing.

Variable	Average	Standard deviation	Multiple comparisons
Learning requirements (1)	3.49	0.03	1 > 2**, 1 > 3***
Emotional preference (2)	3.42	0.03	2 < 1**, 2 > 3***
Ability support (3)	2.84	0.04	3 < 1***, 3 < 2***
Teacher’s expected total score	3.34	0.63	
The synthesis of multiple comparison results			3 < 2 < 1

#### 4.1.2 The impact of background variables on teacher expectations among junior high school students

##### 4.1.2.1 The influence of background variables of middle school students on the total expected score of teachers

**Figure 1:**
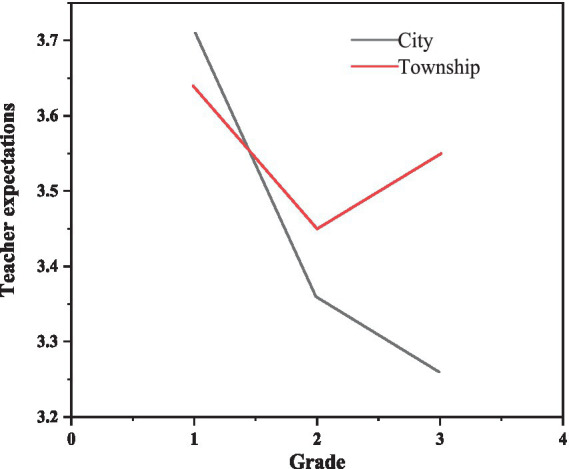
The interactive effect of grade and regional factors on teacher expectations.

The expected total score of teachers in each group is shown in Table 4. Perform a multiple factor analysis of variance with the total expected score of teachers as the dependent variable and gender (male, female), grade (first, second, third year of junior high school), and region (city, township) as independent variables. However, there was a significant interaction between grade levels and regions. The interaction between grades and regions is shown in Figure 1, and the expectations of teachers from different grades and regions are described in Table 5. A simple effect comparison shows that the teacher expectations of urban second and third year students are significantly lower than those of first year students, and the difference between second and third year students is not significant; in rural areas, teacher expectations for second-year junior high school students are notably lower compared to those for first-year students. However, no significant differences were found between teacher expectations for first-year and third-year students, or between second-year and third-year students (Utami and Vioreza, 2021).

**Table 4:** Summary of score description and analysis of variance for total teacher expectations in each group.

Project	Number of people	Average	Standard deviation	*F*
Gender				0.01
Male (1)	294	3.37	0.04	
Girls (2)	289	3.37	0.04	
Grade				11.08***
First grade of junior high school (3)	171	3.56	0.05	
Second grade of junior high school (4)	196	3.26	0.05	
Third grade of junior high school (5)	216	3.39	0.04	
Area				3.23
City (6)	318	3.33	0.04	
Township (7)	265	3.42	0.04	
Gender * Grade				2.76
Gender * Region				1.10
Grade * Region				4.31*
Gender * Grade * Region				1.975

*p < 0.05, ***p < 0.001.

**Table 5:** Description of expected scores for teachers in different regions and grades.

Region	First grade of junior high school		Second grade of junior high school		Third grade of junior high school		*F*
	*M*	SD	*M*	SD	*M*	SD	
City	3.61	0.42	3.22	0.58	3.14	0.54	10.706***
Township	3.61	0.41	3.30	0.53	3.43	0.48	3.01*

*p < 0.05, ***p < 0.001.

##### 4.1.2.2 The influence of background variables of middle school students on various dimensions of teacher expectations

Perform a multivariate analysis of variance with three dimensions of teacher expectations as the dependent variables and gender (male, female), grade (first, second, and third year of junior high school), and region (city, township) as the independent variables (Amunga et al., 2020). The descriptive statistics and analysis of variance of each group in various dimensions of teacher expectations are summarized in Tables 6, 7.

**Table 6:** Description of scores for teacher expectations in various dimensions by gender, grade, and region.

Variation source	*N*	Learning requirements		Emotional preference		Ability support	
		*M*	SD	*M*	SD	*M*	SD
Gender							
Male (1)	294	3.52	0.61	3.36	0.67	2.84	0.78
Girls (2)	289	3.47	0.56	3.5	0.58	2.83	0.78
Grade							
First grade of junior high school (3)	171	3.73	0.46	3.65	0.50	3.04	0.74
Second grade of junior high school (4)	196	3.43	0.63	3.32	0.65	2.67	0.82
Third grade of junior high school (5)	216	3.36	0.6	3.33	0.64	3.01	0.75
Area							
City (6)	318	3.40	0.64	3.34	0.63	2.8	0.80
Township (7)	265	3.60	0.51	3.52	0.61	3	0.74

**Table 7:** Summary of analysis of variance on the effects of gender, grade, and regional factors on teacher expectations in various dimensions.

Variation source	Compare types		Learning requirements	Emotional preference	Ability support
Gender	Wilks’ Λ	0.968**			
	*F*		0.76	3.5	0.34
	Gender comparison		–	–	–
Grade	Wilks’ Λ	0.912***			
	*F*		11.81***	10.12***	6.46**
	Comparison between grades		3 > 4, 5	3 > 4, 5	4 < 3, 5
Area	Wilks’ Λ	0.98			
	*F*		4.506*	2.508	0.140
	Regional comparison		7 > 6	–	–

*p < 0.05, **p < 0.01, ***p < 0.001.

The overall test results of analysis of variance show that gender and grade factors have significant overall main effects on various dimensions of teacher expectations (Wilks’ *Λ* = 0.968, *p* < 0.01; Wilks’ Λ = 0.912, *p* < 0.001), while regional factors have no significant overall main effects (Wilks’ Λ = 0.990, *p* > 0.05). However, grade level significantly impacted all three dimensions, with second and third-year students receiving lower scores in learning requirements and emotional preferences compared to first-year students. Additionally, in terms of ability support, second-year students scored lower than first-year students (Molla and Nolan, 2020). The influence of regional factors on teacher expectations is only reflected in the aspect of learning requirements, and urban students perceive that teachers have lower learning requirements than rural students.

### 4.2 Characteristics of achievement motivation among junior high school students

#### 4.2.1 General characteristics of achievement motivation among junior high school students

Table 8 displays the scores of different components of achievement motivation for junior high school students. By examining the total achievement motivation score along with the mean and standard deviation of each component, it is evident that students’ drive to pursue success outweighs their inclination to avoid failure (Saloviita, 2020). A repeated analysis of variance was conducted on the two dimensions of achievement motivation, and it was found that the overall differences between the two components of achievement motivation were significant (Wilks’ *Λ* = 0.692, *p* < 0.001).

**Table 8:** Description of achievement motivation among junior high school students.

Dimension	Average	Standard deviation
Total score for pursuing success	43.01	0.217
Total score for avoiding failure	35.12	0.29
Total score of achievement motivation	7.78	11.73

#### 4.2.2 The impact of background variables on achievement motivation among junior high school students

**Figure 2:**
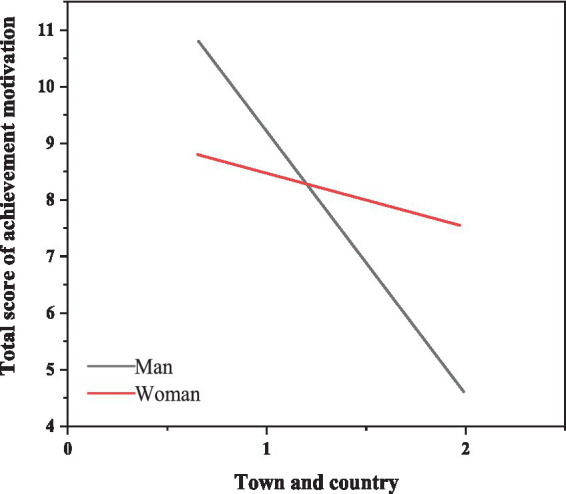
The interaction of gender and regional factors on achievement motivation.

A multivariate analysis of variance examined the relationship between achievement motivation scores among junior high school students and various factors including gender, grade, and region. However, the interaction between gender and region was significant, as illustrated in Table 9 and Figure 2. Further examination in Table 10 revealed that urban boys exhibited significantly higher achievement motivation compared to rural boys. Conversely, no significant difference was found between achievement motivation scores for urban girls and rural girls (Madigan and Curran, 2021).

**Table 9:** Summary table of score description and analysis of variance for total achievement motivation in each group.

Project	Number of people	Average	Standard deviation	*F*
Gender				0.01
Male (1)	294	7.65	0.60	
Girls (2)	289	7.76	0.64	
Grade				0.411
First grade of junior high school (3)	171	7.23	0.83	
Second grade of junior high school (4)	196	8.36	0.84	
Third grade of junior high school (5)	216	7.52	0.70	
Area				13.05***
City (6)	318	9.56	0.6	
Township (7)	265	5.85	0.65	
Gender * Grade				0.7
Gender * Region				5.10*
Grade * Region				1.78
Gender * Grade * Region				1.147

*p < 0.05, ***p < 0.001.

**Table 10:** Description of the total score of achievement motivation among students of different genders and regions.

Gender	City		Township		*F*
	*M*	SD	*M*	SD	
Boys	10.51	12.60	4.45	10.78	18.81***
Girls	8.60	12.00	6.84	10.52	1.70

***p < 0.001.

### 4.3 Analysis of the correlation between teacher expectations, student achievement motivation, academic achievement, and creativity

Calculate the correlation coefficients between observed variables and latent variables separately, as shown in Tables 11, 12. This analysis aims to verify the following hypotheses:

**Table 11:** Correlation coefficients between observed variables.

Variables	1	2	3	4	5	6	7	8	9	10	11
1	1										
2	0.61***	1									
3	0.50***	0.54***	1								
4	0.36***	0.28***	0.37***	1							
5	−0.18***	−0.20**	−0.23**	−0.40**	1						
6	0.11**	0.13***	0.13**	0.18***	0.11**	1					
7	0.00*	0.14***	0.15***	0.20***	0.11**	0.86***	1				
8	0.09*	0.11**	0.13**	0.24***	0.13**	0.78***	0.78***	1			
9	0.18***	0.24***	0.26***	0.20***	−0.20***	0.28***	0.33***	0.284***	1		
10	0.15***	0.21***	0.24***	0.25***	−0.27***	0.26***	0.32***	0.27***	0.70***	1	
11	0.18***	0.24***	0.28***	0.26***	−0.21***	0.25***	0.32***	0.25***	0.73***	0.80***	1

Observed variable numbers correspond to: 1 = learning requirements, 2 = emotional preferences, 3 = ability support, 4 = pursuit of success, 5 = avoidance of failure, 6 = fluency, 7 = flexibility, 8 = originality, 9 = Chinese scores, 10 = mathematics scores, 11 = English scores. Correlation coefficients are based on Pearson two-tailed tests, *p < 0.05, **p < 0.01, ***p < 0.001.

**Table 12:** Correlation coefficients between latent variables.

Latent variable	1	2	3	4
1 Teacher expectations	1			
2 Achievement motivation	0.38***	1		
3 Creativity	0.16***	0.22*	1	
4 Academic achievements	0.28***	0.31***	0.34***	1

*p < 0.05, **p < 0.01.

*H1*: The three dimensions of teacher expectations (learning requirements/emotional preferences/ability support) are positively correlated with academic achievement.

*H2*: Achievement motivation (pursuing success/avoiding failure) mediates the relationship between teacher expectations and academic achievement.

*H3*: There is a weak correlation between creativity indicators and teacher expectations, but they are moderated by achievement motivation.

Table 11 illustrates several significant correlations among various factors. Specifically, learning requirements demonstrate a strong positive correlation with emotional preference, ability support, pursuit of success, Chinese language proficiency, mathematics proficiency, and English proficiency at the 0.001 level. Additionally, there are positive correlations with fluency at the 0.01 level, flexibility, and originality at the 0.05 level, and a significant negative correlation with avoiding failure at the 0.001 level. Moreover, ability support is significantly positively correlated with pursuit of success, fluency, flexibility, Chinese language proficiency, mathematics proficiency, and English proficiency at the 0.001 level. It also shows a positive correlation with originality at the 0.01 level and a negative correlation with avoiding failure at the 0.01 level. The pursuit of success demonstrates significant positive correlations with fluency, flexibility, originality, Chinese language proficiency, mathematics proficiency, and English proficiency at the 0.001 level, and a negative correlation with avoiding failure at the 0.01 level. Avoiding failure exhibits significant negative correlations with Chinese, mathematics, and English grades at the 0.001 level, as well as fluency, flexibility, and originality at the 0.01 level. Furthermore, fluency shows significant positive correlations with flexibility, originality, Chinese language proficiency, mathematics proficiency, and English proficiency at the 0.001 level. Flexibility exhibits significant positive correlations with originality, Chinese language proficiency, mathematics proficiency, and English proficiency at the 0.001 level. Lastly, originality demonstrates significant positive correlations with Chinese, mathematics, and English grades at the 0.001 level, while Chinese language proficiency, mathematics proficiency, and English proficiency show significant positive correlations at the 0.001 level. Preliminary correlation analysis supports the pathway through which teacher expectations influence academic achievement through achievement motivation (H1–H2), while creativity may be influenced through indirect pathways (H3), and the specific mechanism needs to be verified through subsequent structural equation modeling. Ensure comparability between groups through random grouping and baseline balance testing (all *p* > 0.20). The significant increase in teacher expectations in the intervention group (*β* = 0.79, *p* < 0.001) confirmed the validity of experimental manipulation. The township experimental group showed a greater improvement in academic achievement (β – diff = 1.62, *p* = 0.032), supporting the hypothesis of marginal effect enhancement of teacher expectations in an environment of scarce educational resources. Despite using a controlled experimental design, the expected transmission process of teachers may still be influenced by unmeasured classroom interaction quality. It is recommended to add classroom video encoding as a process variable in future research.

The latent variable correlation coefficients in Table 12 show that teacher expectations are significantly positively correlated with academic performance (*r* = 0.28, *p* < 0.001) and weakly correlated with creativity (*r* = 0.16, *p* < 0.001). Achievement motivation partially mediates the effect of teacher expectations on academic performance (*r* = 0.31, *p* < 0.001) and fully mediates its effect on creativity (*r* = 0.22, *p* < 0.05).

### 4.4 The relationship between teacher expectations, student achievement motivation, academic achievement, and creativity

Based on experimental grouping data, this section validates the hypothesis path through structural equation modeling. The baseline balance of the high/low expectations group has been tested (all variables *p* > 0.20) to ensure the validity of subsequent causal inference. From the above results analysis, there is a noticeable correlation observed between teacher expectations, student achievement motivation, academic achievement, and creativity. In James’s framework, when the influence of the independent variable *X* on the dependent variable *Y* occurs through an intermediary variable *M*, *M* is referred to as the mediator variable. In this study, the author employed a mediation analysis approach, with teacher expectations serving as the independent variable, achievement motivation as the mediator, and academic achievement and creativity as the dependent variables. The AMOS 24.0 software package was utilized, employing the maximum likelihood estimation method to assess how well the model fits the data. This analysis aimed to confirm whether achievement motivation mediates the relationship between teacher expectations and academic outcomes (Botes et al., 2020; Peng and Kievit, 2020).

#### 4.4.1 The relationship between teacher expectations, achievement motivation and academic performance under experimental design

**Figure 3:**
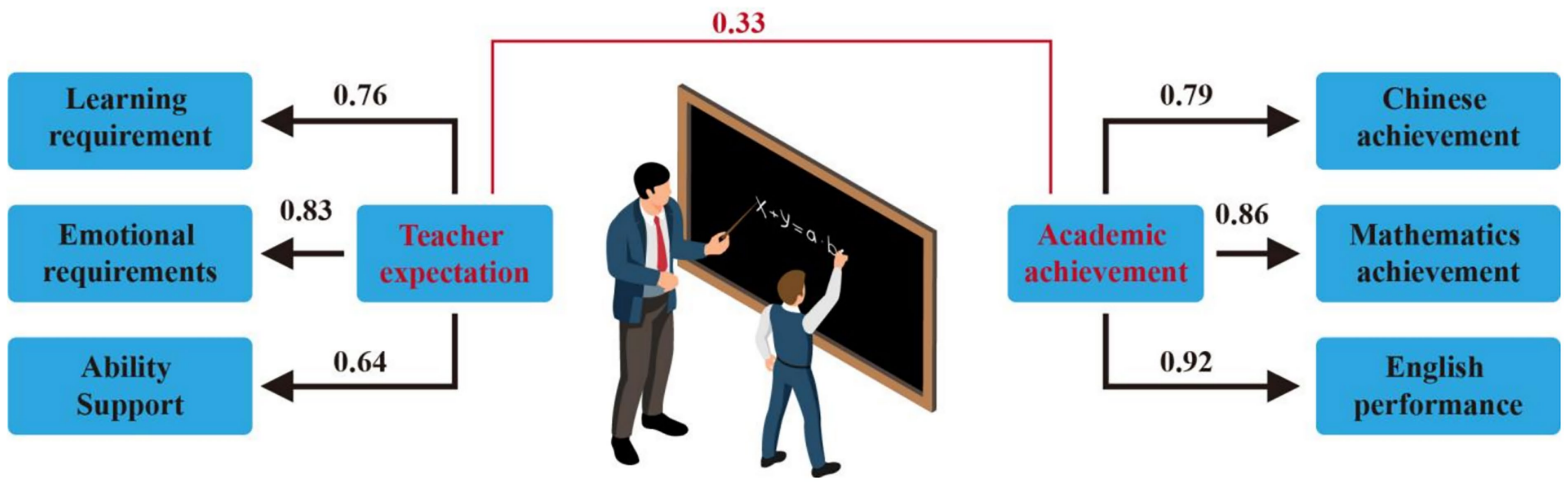
The relationship between teacher expectations and student academic achievement.

To begin with, the analysis focuses on teacher expectations as the independent variable and assesses their influence on student academic achievement. The objective is to determine whether the coefficient *c* holds significance. Figure 3 illustrates the detailed analysis results of this model, indicating a positive effect of teacher expectations on student academic achievement. The standardized regression coefficient c is noted as 0.33, signifying significance at the 0.001 level.

**Figure 4:**
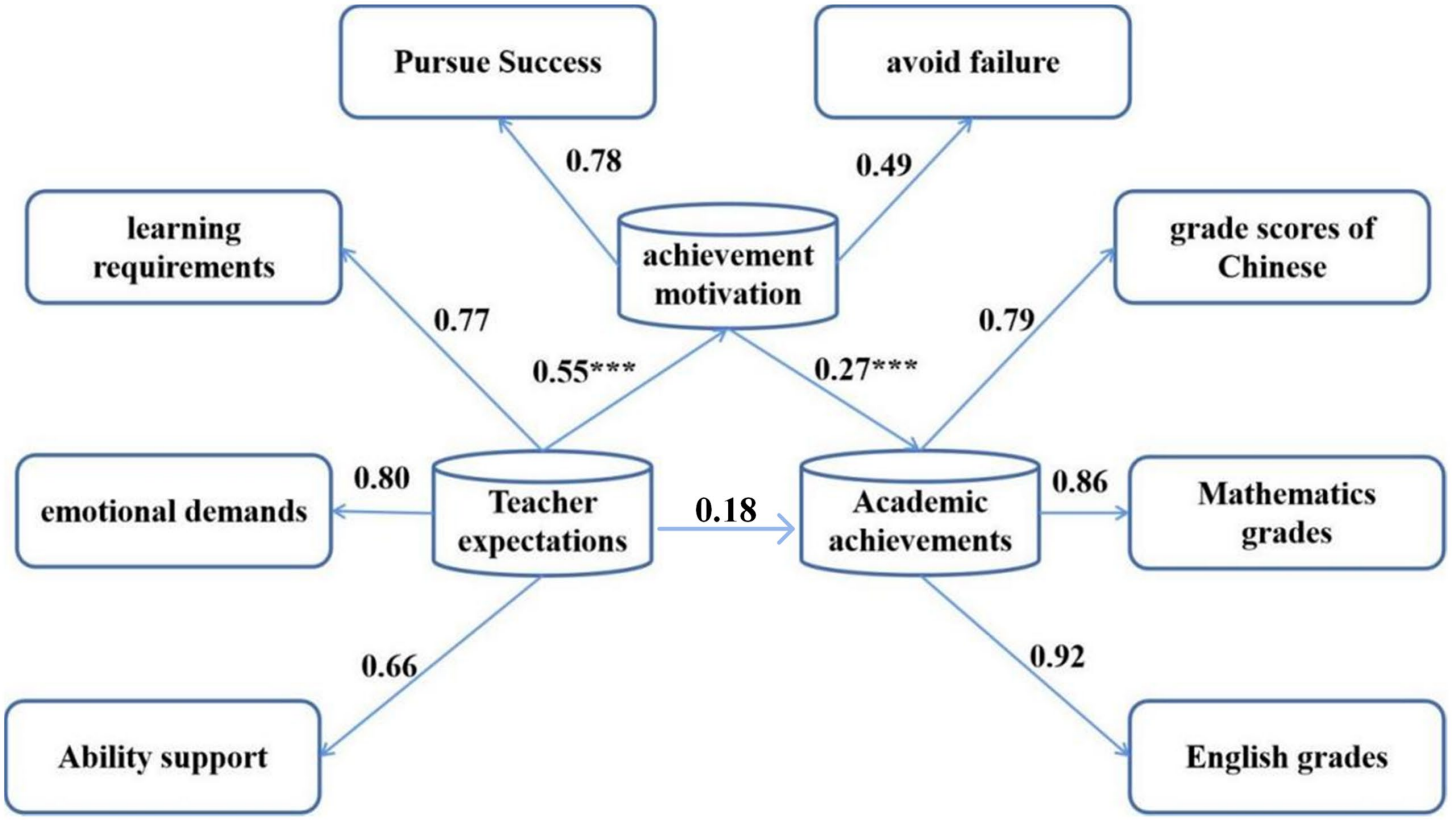
The mediating role of achievement motivation in the relationship between teacher expectations and student academic achievement.

After introducing achievement motivation as a mediating variable into the structural equation model, the significance of coefficients *a* and *b* was tested (Pinquart and Ebeling, 2020; Abuhassna et al., 2020). The results of the analysis are depicted in Figure 4. Where *a* is the path coefficient of teacher expectations to achievement motivation, and b is the path coefficient of achievement motivation to academic achievement.

This paper proceeded to assess the significance of the coefficient *c*’ (The direct effects of teacher expectations on academic achievement). It was observed that *c*’ decreased to 0.18; however, it remained significant at the 0.01 level.

From the data analysis results of the model shown in Figure 4, it can be seen that the direct effect of teacher expectations on student academic achievement is *c*’=0.18, the indirect effect is *a* * *b* = 0.55 * 0.27 = 0.1485, the total effect is 0.3285 (*a* * *b* + *c*’). The fitting indexes of this model are shown in Table 13.

**Table 13:** Various fitting indices of the mediation model.

$\chi^{2}$	df	$\chi^{2}$ /df	RMSEA	GFI	AGFI	NFI	IFI	CFI	TLI
54.211	17	3.188	0.051	0.966	0.940	0.962	0.970	0.970	0.958

From the various fitting indices of the model, it can be seen that the theoretical model fits well with the data, indicating that the mediation effect model has high stability.

## 5 Discussion

### 5.1 The relationship between teacher expectations, student achievement motivation, and creativity

Firstly, using teacher expectations as the independent variable, examine their impact on creativity, that is test whether the coefficient *c* is significant. Teacher expectations exhibit a positive influence on creativity, indicated by a standardized regression coefficient of 0.16, which is significant at the 0.001 level. Subsequently, achievement motivation is included as a mediating variable in the structural equation model to evaluate the significance of coefficients *a* and *b*. In other words, after further testing, we observed that the coefficient *c*’ decreased to 0.03, indicating no significant difference (Shana and Abulibdeh, 2020).

The direct effect of teacher expectations on student creativity is *c*’ = 0.03, the indirect effect is *a* * *b* = 0.54 * 0.24 = 0.130, the total effect is 0.150 (*a* * *b* + *c*’), looking at the various fitting indices of the model, it’s evident that the theoretical model aligns well with the data. Additionally, the mediating effect explains 81.14% of the total effect, highlighting the robustness of the model in explaining the relationship between variables, indicating that the mediation effect model has high stability (Türel and Dokumaci, 2022).

### 5.2 The relationship between teacher expectations, student achievement motivation, academic achievement, and creativity

Upon integrating teacher expectations, student achievement motivation, academic achievement, and creativity into the model, it emerged that the influence of teacher expectations on creativity was not statistically significant (*p* > 0.05). However, teacher expectations significantly and positively affected both achievement motivation and academic achievement (*p* < 0.001). From the various fitting indices of the model, it can be seen that the theoretical model fits well with the data, indicating that the model has high stability (Liu et al., 2020).

In the study, teacher expectations had a significant impact on academic achievement through achievement motivation. The mechanism of this path is reflected in the fact that teachers’ high expectations enhanced students’ motivation to pursue success, which in turn improved their performance in Chinese, mathematics and English exams. The standardized regression coefficient showed that the direct effect was 0.18, and the indirect effect reached 0.1485 through achievement motivation, indicating that achievement motivation played a partial mediating role in this relationship. Data analysis also revealed that the direct effect of teacher expectations on academic achievement coexisted with the indirect effect through achievement motivation. The significance of the direct path indicated that teacher expectations may directly shape students’ learning performance through classroom behavior or feedback, while the indirect path further strengthened this effect by stimulating students’ intrinsic motivation. In contrast, the impact of teacher expectations on creativity was completely dependent on the mediation of achievement motivation, with an indirect effect of 0.13, while the direct effect dropped to 0.03 and was not significant. This result suggests that the improvement of creativity depends on the activation of students’ intrinsic motivation, rather than the direct effect of teacher expectations. In the experimental design, the difference in academic achievement between the high expectation group and the low expectation group was *β* = 0.32, *p* < 0.001, indicating that the standardized test scores of students in the high expectation group were significantly higher than those in the low expectation group, while the difference in creativity indicators was manifested through the mediating effect of achievement motivation. The academic achievement of students in township schools under high expectation conditions increased more significantly, with a β difference value of 1.62, *p* = 0.032, which is consistent with the hypothesis of enhanced marginal effects in an environment of scarce educational resources.

## 6 Conclusion

This study verified the psychological effect of teacher expectations on the academic achievement and creativity of junior high school students through a controlled experimental design. In the experiment, teachers were randomly assigned to a high expectation group and a low expectation group. Teachers in the high expectation group were told that their students had high learning potential, while teachers in the low expectation group were told that their students had low potential. This intervention was implemented by providing false information about students’ abilities to teachers and lasted for one semester. Achievement motivation partially mediated the relationship between teacher expectations and academic achievement, with a mediation ratio of 45.2%, an indirect effect of 0.15, and a 95% confidence interval of 0.09–0.21. The effect of teacher expectations on creativity was completely mediated by achievement motivation, with an indirect effect of 0.13, a 95% confidence interval of 0.07–0.23, and a non-significant direct effect, *β* = 0.03. This design directly verifies the causal effect by manipulating the level of teacher expectations and controlling other teaching variables, providing empirical evidence for educational practice.

The limitations of this study are reflected in the following aspects. Despite the use of a controlled experimental design, the transmission process of teacher expectations may still be affected by the unmeasured quality of classroom interaction, such as teachers’ non-verbal behavior or interaction patterns between students, which may partially explain the differences between high and low expectation groups. This study only selected junior high schools in cities and towns as research objects, and the geographical and educational background of the sample limits the generalizability of the results to other educational stages or regions. In addition, this study focuses on academic achievement and creativity as outcome variables, and does not measure other student characteristics that may be affected by teacher expectations, such as self-esteem or self-efficacy. The omission of these variables may limit the comprehensive understanding of the effect of teacher expectations.

The results of this study have important implications for educational policy and practice. Education policy makers need to pay attention to the positive impact of teacher expectations on students’ academic achievement and creativity, and encourage teachers to raise their expectations for students by formulating relevant policies, such as incorporating expectation management into the teacher evaluation system. School administrators need to strengthen teacher training to improve their ability to regulate student expectations, and reduce low-expectation behaviors caused by subjective bias through workshops or case analysis. In daily teaching, teachers need to be aware of the role of high expectations in promoting student motivation and performance, and stimulate students’ enthusiasm and potential for learning through positive feedback and creative task design, thereby promoting their all-round development.

## Data Availability

The original contributions presented in the study are included in the article/supplementary material, further inquiries can be directed to the corresponding author.
